# Tunneling Atomic Layer-Deposited Aluminum Oxide: a Correlated Structural/Electrical Performance Study for the Surface Passivation of Silicon Junctions

**DOI:** 10.1186/s11671-019-3160-2

**Published:** 2019-10-22

**Authors:** Kangping Liu, Odile Cristini-Robbe, Omar Ibrahim Elmi, Shuang Long Wang, Bin Wei, Ingsong Yu, Xavier Portier, Fabrice Gourbilleau, Didier Stiévenard, Tao Xu

**Affiliations:** 1 Key Laboratory of Advanced Display and System Application, Shanghai University, Shanghai, 200072 China; 2 PHLAM, UMR8523, Université de Lille 1, 59652 Villeneuve d’Asq Cédex, France; 3 Université de Djibouti, Groupe de Recherche PCM, Faculté des Sciences, BP 1904, Djibouti City, Djibouti; 4 Department of Materials Science and Engineering, National Dong Hwa University, No. 1, Sec. 2, Da Hsueh Rd. Shoufeng, Hualien, 97401 Taiwan, Republic of China; 5 CIMAP, Normandie Univ, ENSICAEN, UNICAEN, CEA, CNRS, 6 Boulevard Maréchal Juin, 14050 Caen Cedex 4, France; 6 IEMN, UMR8520, Université de Lille 1, 59652 Villeneuve d’Ascq Cédex, France

**Keywords:** Surface passivation, Atomic layer deposition, Alumina layer, Structural/electrical properties, Silicon p-n junction

## Abstract

Passivation is a key process for the optimization of silicon p-n junctions. Among the different technologies used to passivate the surface and contact interfaces, alumina is widely used. One key parameter is the thickness of the passivation layer that is commonly deposited using atomic layer deposition (ALD) technique. This paper aims at presenting correlated structural/electrical studies for the passivation effect of alumina on Si junctions to obtain optimal thickness of alumina passivation layer. High-resolution transmission electron microscope (HRTEM) observations coupled with energy dispersive X-ray (EDX) measurements are used to determine the thickness of alumina at atomic scale. The correlated electrical parameters are measured with both solar simulator and Sinton’s *Suns-Voc* measurements. Finally, an optimum alumina thickness of 1.2 nm is thus evidenced.

## Introduction

The reduction in surface recombination losses in silicon p-n junctions is of prime importance in order to improve the efficiency of light absorption and its conversion into photocurrent, with solar cells as one main application [1, 2]. Among the key process that can improve the defect recombination in silicon junctions, the passivation of the surface and the contacts were and are always of prime importance. Front and rear surface passivations have been developed, both for the illuminated non-metallized regions as well as for the metal silicon contacts [3, 4]. The metal-silicon interface features large recombination, so two options have been developed to minimize the losses at the contact area: small contact area associated with low local doping level, or local passivation of the metal-silicon interface by the introduction of a thin tunneling dielectric layer. Recently, a new route with a promising potential has been suggested using a carrier-selective passivation layer [5]. In this case, one polarity of charge carriers is allowed to pass to the metal whereas the other polarity is blocked.

Among all the passivation layers, aluminum oxide (Al_2_O_3_) deposited by atomic layer deposition (ALD) is one of the most used methods, even if plasma-enhanced chemical vapor deposition (PECVD) process can be also applied [6, 7]. ALD allows a good control of the thickness down to atomic scale, while the use of alumina leads to a good chemical passivation of interface states as well as to an efficient field effect passivation through localized charges in the oxide layer [8]. For example, Elmi et al. showed that the introduction of embedded Ag nanoparticles in a thin alumina layer can effectively enhance the field effect passivation [9]. It is known that the sign and the density of the localized charges as well as the thickness of alumina layer are important parameters for surface passivation. Many works have been published to study the influence of alumina thickness on the device performance; however, there is no consensus on the optimal alumina thickness since it varies from 0.24 to 30 nm in the literature, as it is summarized in Table 1. Table 1 illustrates the scattering of the optimized alumina thickness. In fact, many data concern the surface recombination velocity (Se) which does not correspond to measurements on the fabricated solar devices. The general trend is a decrease of Se for larger thickness values due to a better chemical passivation by the presence of hydrogen in the alumina layer which passivates interface states during the post-growth thermal annealing.

**Table 1:** Summary of alumina passivation effects on silicon p-n junctions

Work’s reference	Technique of deposition	Temperature (°C)	Type of materials	Thermal treatment	Optimized thickness	Physical parameters
Hoex et al. [10]	Plasma-assisted (PA) ALD	200	p-type, 2.0 Ω·cm n-type, 1.9 Ω·cm	30 min, 425 °C, N_2_	7 nm	Se < 5 cm/s on n- and p-types
Hoex et al. [11]	PA ALD	200	n-type, 1.9 Ω·cm	30 min, 425 °C, N_2_	6–32 nm	Life time *τ*_e_ 0.4 to 1 ms
Schmidt et al .[12]	PA ALD + 75 nm PECVD SiN_x_	200	p-type, 1.5 Ω·cm	30 min, 425 °C, N_2_	3.6 nm	Se < 22 cm/s
Dingemans et al. [13]	PA ALD + 70 nm PECVD SiN_x_	200	n-type, 2 Ω·cm	425 °C, 30 min, N_2_	30 nm	Se < 3 cm/s
Terlinden et al. [14]	PA ALD	200	p-type, 2 Ω·cm	400 °C, 10 min, N_2_	5–20 nm 2–5 nm	Se = 20 cm/s Se increases up to 70 cm/s
Dingemans et al. [15]	PA ALD	200	n-type, 3.5 Ω·cm	425 ± 50 °C, 30 min, N_2_	5–30 nm < 5 nm	Se_min_ = 0.8 cm/s Se_min_ = 2.5 cm/s
	Thermal ALD	200	n-type, 3.5 Ω·cm p-type, 2.2 Ω·cm	375 ± 50 °C	10–30 nm < 10 nm	n-type: Se_min_ = 2 cm/s p-type: Se_min_ = 3–4 cm/s
Werner et al. [16]	Thermal ALD	200	p-type, 1.3 Ω·cm	425 °C, 15 min, N_2_	> 10 nm	Se < 200 cm/s
Richter et al. [17]	PA ALD + 70 nm PECVD SiN_x_	230	p-type, 1 Ω·cm	350–450 °C, 10 min, N_2_	0.5–3 nm	Se = 40 cm/s, *τ*_e_ = 1 ms, emitter saturation current Joe = 30 fA/cm^2^
Zielke et al. [18]	PA ALD	200	n^+^	425 °C, 15 min, N_2_	0.24 nm	PCE = 21%, Joe = 174 fA/cm^2^
Garcia-Alonso et al. [19]	PA ALD	200	n-type, 3.5 Ω·cm p-type, 2.5 Ω·cm	400 °C, 5–10 min, N_2_	1–2 nm > 3 nm	Se = 100–700 cm/s Se < 4 cm/s
Kotipalli et al. [20]	PA and thermal ALD + PECVD SiO_2_ (20 nm) or SiNx (20 nm)	250	p-type, 1–3 Ω·cm	432 °C, 30 min, N_2_/H_2_	15 nm	Se = 3 cm/s
Albadri [21]	PA ALD	200	p-type, 13 Ω·cm	400 °C, 30 min, N_2_	20 nm	Se = 15 cm/s
Deckers et al. [22]	Thermal ALD	200	n-type, 0.8–5 Ω·cm p-type, 2 Ω·cm	500 °C, 30 min, N_2_	25 cycles	Life time 400 μs, for n- and p-type
van de Loo et al. [23]	PA ALD for alumina and SiO_2_ + 70 nm PECVD SiNx	200	p^+^ and n^+^	400 °C, 10 min, N_2_	SiO_2_ 0–14 nm Alumina 30 nm	For n^+^, Joe = 50 fA/cm^2^ For p^+^, Joe < 54 fA/cm^2^

Richter et al. reported that emitter saturation current down to 30 fA/cm^2^ could be obtained for thin layer (0.5 to 3 nm) but with a stack structure constituted of alumina and 70 nm of SiN_x_ [17]. The best metal-silicon passivated contact is observed with a 0.24-nm-thick Al_2_O_3_ [18]. Finally, concerning the doping and type levels, passivation is more efficient on n^+^ but a SiO_2_/alumina stack structure gives the possibility to tune the density of localized charges and can be used on both n- or p-type [23]. Nevertheless, only few works demonstrated the correlation between the quality of the substrate/alumina interface and the electrical performances of devices. It is thus necessary to perform a systematic observation at atomic scale on the alumina passivation layer and to obtain an optimize value of the alumina thickness correlated with electrical performances.

In this paper, alumina layers deposited by using the ALD technique with different thicknesses from 0.24 to 1.9 nm were used to passivate implanted Si n^+^-p junctions. The interface of alumina layer on the front surface of Si junction was studied by using high-resolution transmission electron microscope (HRTEM), while the thickness of alumina layer was correlated to the associated electrical parameters such as serial resistance, ideality factor, lifetime, external quantum efficiency (EQE), and power conversion efficiency (PCE). Sinton’s *Suns-Voc* measurements have been performed to resolve the influence of series resistance. Finally, an optimized 1.2 nm alumina thickness was obtained. It should be noted that we focus here only on the front contact passivation of silicon p-n junctions; the device efficiency is not fully optimized which is out of purpose of this work.

## Methods

### Device Fabrication

**Fig. 1:**
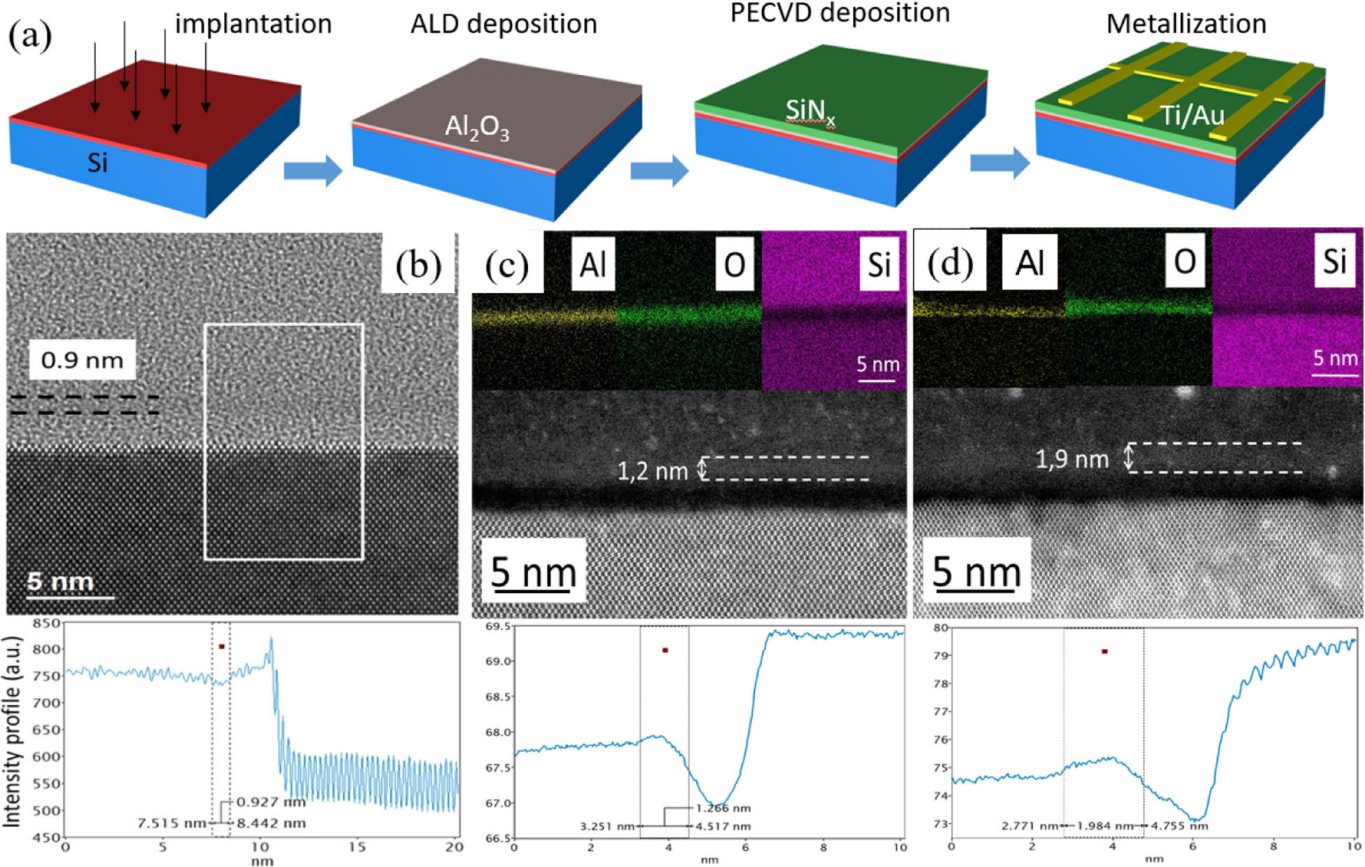
**a** Schematic of fabrication process of implanted Si n^+^-p junction passivated by Al_2_O_3_/SiN_x_ stack. **b** HRTEM image taken along the [011] direction of the silicon substrate. Intensity profile corresponding to the white rectangle (an alumina film of a thickness about 0.9 nm is visible on the top of the silica layer). **c**, **d** STEM HAADF images of the two alumina layers grown by ALD with the corresponding STEM EDX maps of Al, O, and Si. The brighter contrasts in the HAADF images on the top of the silica layer are due to the higher density (higher average *Z* value) compared to that of silica or silicon nitride. Intensity profiles give the thicknesses of alumina layers of ~ 1.2 nm and 1.9 nm, respectively

Figure 1a shows the fabrication process of implanted Si n^+^-p junctions with surface passivation of Al_2_O_3_/SiN_x_:H stacks. Four-inch boron-doped p-type silicon (100) wafers with a resistivity of 5–10 Ω·cm were used as substrates. The samples were cleaned using piranha solution and distilled water before the realization of n^+^ top layer. Phosphorous ion implantation was performed using a dose of 10^14^ at/cm^2^ at 180 keV, followed by an annealing at 900 °C during 5 min to activate the dopants. Detailed descriptions of the technological process can be found in our previous works [24, 25].

An ultrathin Al_2_O_3_ tunneling layer with an expected thickness *d* ranging from 0.24 to 1.9 nm was deposited by using the ALD technique. Note that this thickness has been deduced from the average thickness determined by ellipsometry spectroscopy, obtained for alumina thin films grown with different number of cycles. One ALD cycle deposited one monolayer which equals to 0.12 nm. Junctions without alumina (*d* = 0), i.e., with unpassivated metal-silicon contacts, have been realized, but the PCE is very low, only 0.4% [9]. The alumina deposition was carried out in a PICOSUN R200 system through a thermal process. The reactants used were trimethylaluminum (TMA) and H_2_O, while the growth temperature was 290 °C. During the ALD process, water cycles were used to oxidize the TMA precursor. As a result, a thin SiO_2_ oxide layer was deposited between the silicon surface and the alumina layer due to the natural oxidation of the silicon surface. The thickness of such native oxide layer observed by TEM was close to 1.5 nm. Second, an 80-nm-thick SiN_x_:H layer which corresponds to the value usually used in Si-solar cell industry was deposited on the sample by using the PECVD approach with a mixture of SiH_4_ and NH_3_. The deposition temperature was 340 °C, while the pressure was 1 Torr and the power was 10 W. Sample was then annealed at 650 °C for 10 min to make H diffusing into Si.

Finger electrodes of Ti/Au (20/800 nm) were deposited on the front side by sputtering with a shadow mask after the opening of the SiN_x_:H coating by using reactive ion etching (RIE). The back contact was then deposited by evaporating a 400-nm-thick Ti/Au film. Finally, the samples were annealed at 400 °C for 10 min to form ohmic contact.

### Characterization

The TEM analysis was performed from cross-sectional thin foils prepared by focused ion beam (FIB) on a FEI Helios dual-beam Nanolab 600i. Prior to the ion thinning down, a carbon film and a platinum layer were deposited to protect the top surface of the sample. The TEM, STEM high-angle annular dark field (HAADF), and STEM energy dispersive X-ray (EDX) observations were done with a double corrected JEOL ARM200F cold FEG microscope operated at 200 kV and equipped with an EDX spectrometer (CENTURION from JEOL). The image processing was performed using DIGITALMICROGRAPH (GATAN). The images were taken with the electron beam parallel to the [011] direction of the Si (100)-oriented substrate. In this orientation, the electron beam is parallel to the alumina/substrate interface.

The electrical parameters under illumination were measured using a solar simulator (Oriel®Sol3ATM) under AM 1.5G illumination, while the external quantum efficiency (EQE) spectra were measured under standard measurement conditions on a 7-SCSpec system manufactured by 7-STAR Co. To overcome the series resistance influence, Sinton’s *Suns-Voc* measurements have been performed [26–28]. Sinton’s *Suns-Voc* technique is an open-circuit method to indicate the performance of a p-n junction or solar cell which allows to compare the electrical parameters given by the solar simulator with the ones deduced without the influence of the series resistance. The setup includes a xenon flashlamp with a full set of neutral-density filters and a wafer stage controlled at 25 °C. A standard I-V curve format with an estimated *Jsc* can be performed by either probing the p+ and n+ regions directly or probing the metallization layer. The data can be used directly to indicate the material and passivation quality of solar cells.

## Results and Discussion

Figure 1(b) is a typical HRTEM image taken along the [011] direction of the silicon substrate. In this direction, the electron beam is necessarily parallel to the film substrate interface. Note that the top surface of the substrate is not perfectly flat. This observation implies that the interfaces between the different above amorphous layers (silica, alumina and silicon nitride) are also rough, making their characterization a very difficult task. Indeed, the thickness measurement is always overestimated due to this roughness. The inset of Fig. 1(b) is an intensity profile perpendicular to the substrate and over a 10 nm wide region as indicated by the white rectangle of the HRTEM image. This profile gives evidence of the difference in contrast between the three amorphous layers on the top of the Si substrate. Indeed, due to the Z contrast, a darker 0.9 nm thick layer can be observed above the silica layer, which is most probably the alumina layer grown by ALD. To confirm this result, high angle annular dark field imaging has been performed on two different alumina layers combined with chemical mapping obtained by scanning transmission electron microscopy coupled with an energy dispersive X ray spectrometer. Figure 1(c) and (d) are two sets of data illustrating two different alumina layer thicknesses.

Both sets are composed of an HAADF image presenting the top surface of the Si substrate (along the [011] direction) and the three amorphous layers namely silica (dark region), alumina (whiter region) and finally silicon nitride (intermediate contrast). Note that some bright dots are visible especially in Fig. 1(d). These features are due to platinum dusts coming from the protection layer during the FIB preparation of the thin foil. For both structures, STEM EDX chemical maps of aluminum, oxygen and silicon are reported on the top of the Fig. 1(c) and (d). The aluminum maps show nicely the presence of aluminum corresponding to the whiter regions of the HAADF images. It is found that some bright dots are present in the adjacent regions but these correspond to some “noise” in the background during acquisition. Wider regions are visible in the oxygen maps since they image the alumina and silica layers. Finally, the silicon maps depict a dark line corresponding to the alumina layers, the only layer without silicon. Due to diffusion scattering phenomena, the chemical maps are not the best data to estimate properly the thickness values.

In order to show more clearly the different contrast induced by the presence of the alumina layer, we have plotted the intensity profiles for both images as shown in Fig. 1(c) and (d). As clearly demonstrated by these profiles, a broad band indicates the brighter regions corresponding to the alumina layer. Considering that the alumina has a certain roughness, it is reasonable to estimate the layer thickness by measuring the distance between two vertical lines located at the middle of the slopes on each side of the layer. The results are about 1.2 nm and 1.9 nm, respectively.

### Evolution of the Series Resistance *R*_s_

**Fig. 2:**
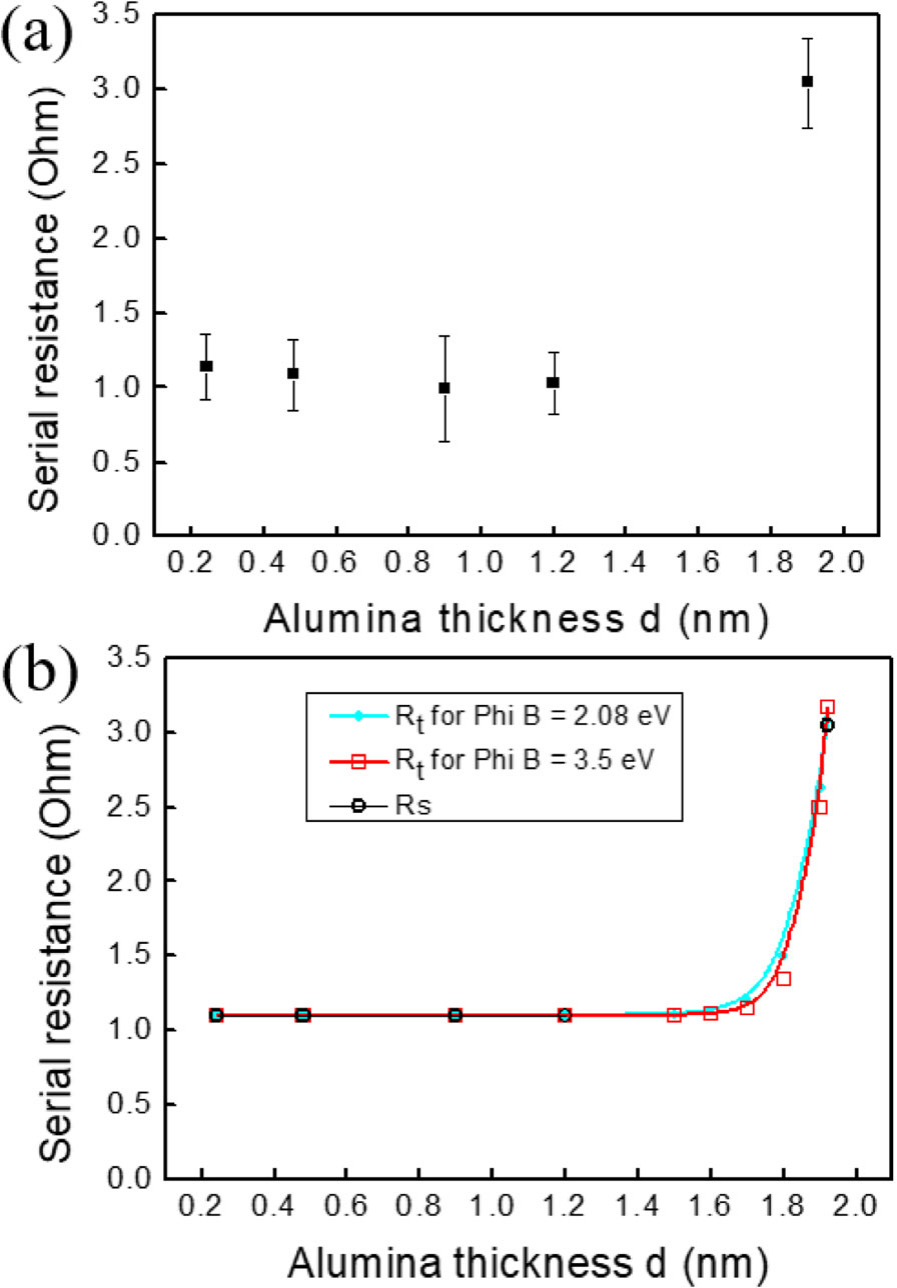
**a** Evolution of the series resistance *R*_s_ versus the alumina thickness. **b** Experimental values of *R*_s_ and simulated one *R*_simu_ calculated for *ϕ*_B_ = 2.08 and 3.5 eV versus the alumina thickness *d* (nm)

As shown in Fig. 2a, *R*_s_ is almost constant (*R*_s_ = 1.1 ± 0.15 Ω) from *d* = 0.24 to 1.2 nm and increases abruptly to 3.1 ± 0.2 Ω for *d* = 1.9 nm. The measured resistance *R*_s_ is the addition of the emitter and base zones, of the metallic fingers, and of the resistance associated with the thin SiO_2_ oxide layer, altogether labeled *R*_i_, plus the resistance *R*_thu_ associated to the alumina layer. For all the samples, in the limit of the reproducibility of the technology procedure given by the error bars (± 0.15 Ω) in Fig. 4, *R*_i_ is considered as constant since the same technological process is applied. *d* is the only modified parameter. So, as *R*_s_ is constant up to *d* = 1.2 nm and as the tunneling resistance *R*_thu_ obviously varies with *d*, we conclude that up to *d* = 1.2 nm, *R*_thu_ varies but its variation is less than the dispersion of the measurements, i.e., 0.15 Ω. *R*_thu_ is directly bound to the inverse of the transfer coefficient γ for tunneling, i.e., the tunneling probability of carriers through a rectangular barrier, given by [29]:

1 $$ \gamma \approx \kern0.5em \exp \left(-\frac{2d\sqrt{2q{m}^{\ast }{\upphi}_{\mathrm{B}}}}{\overline{h}}\right) $$

where m^*^ is the effective mass in the alumina barrier (m^*^ = 0.75 *m*_0_ [30], with *m*_0_ as the electron mass), *h* is the Planck constant, *q* is the electron charge, and *ϕ*_B_ is the effective barrier height, equal to the conduction band offset ΔE_C_ between dielectric and the n^+^ silicon contact. The tunneling resistance is given by:

2 $$ {R}_{\mathrm{thu}}= Ax{\gamma}^{-1} $$

where *A* is a constant. For *d* = 1.9 nm, *R*_thu_ corresponds to the step measured on *R*_s_, and therefore, we deduce *R*_thu_ (1.9 nm) = 2 Ω. From this value, *A* can be calculated. For that, we have to know *ϕ*_B_ which is equal to the conduction band offset between Si and the dielectric layer, since the Fermi level is within the minimum of the conduction band in the heavily doped n^+^ silicon contact. In fact, the dielectric layer is actually a few nanometers SiO_2_/Al_2_O_3_ stack, so the band offset depends on these two dielectric layers. The conduction band offset is in the 3.13–3.5 eV and 2.08–2.8 eV range for SiO_2_ and Al_2_O_3_ [31], respectively. Table 2 gives the values of the *A* prefactor deduced from the value of *R*_s_ measured at *d* = 1.9 nm, for the two extreme values of *ϕ*_B._

**Table 2:** A prefactor deduced from *R*_s_ measured for *d* = 1.9 nm and calculated for the extrema values of *ϕ*_B_ (2.08 and 3.5 eV)

*ϕ*_B_ (eV)	2.08	3.5
*A* (Ω)	1.34 × 10^−11^	6.19 × 10^−15^

In Fig. 2b, we have plotted *R*_s_ and the total simulated resistance *R*_simu_ = *R*_i_ + *R*_thu_ for the two *ϕ*_B_ values versus *d* (*R*_s_ has been taken to its average value 1.1 Ω between *d* = 0.24 and 1.2 nm). Whatever *ϕ*_B_, for *d* ≤ 1.2 nm, *R*_thu_ is negligible. It confirms our starting hypothesis: the step observed at 1.9 nm in the evolution of *R*_s_ is associated with the evolution of the tunneling barrier. For *d* less than 1.2 nm, the main effect of alumina layer is to passivate the n^+^ contacts and the p surface, by means of field effect passivation associated with the fixed charges localized in the oxide. For larger *d* values, the alumina layer introduces a parasitic series contact resistance that decreases the electrical performances of the cells.

Finally, we can estimate the resistivity *ρ* of the alumina layer. For that, we consider the measured resistance *R* for a thickness value of 1.9 nm. The tunneling effect decreases, and the layer begins to have a “bulk-like” behavior (a crude approximation). With such an alumina thickness (*d* = 1.9 nm) and considering the surface *S* of the contact (10.54 mm^2^), we deduce *ρ* using the following equation:

3 $$ R=\rho\ \frac{d}{S} $$

That leads to *ρ* = 1.1 × 10^6^ Ω·cm. For bulk materials on the markets, depending of the growth temperature and of the impurities in the alumina, the resistivity value varies from 10^5^ to 10^14^ Ω·cm (from Kyocera™). So, our estimated value shows that we have a “pseudo-bulk” material, at the limit between a thin layer and a bulk layer.

### Evolution of the Ideality Factor *n*

**Fig. 3:**
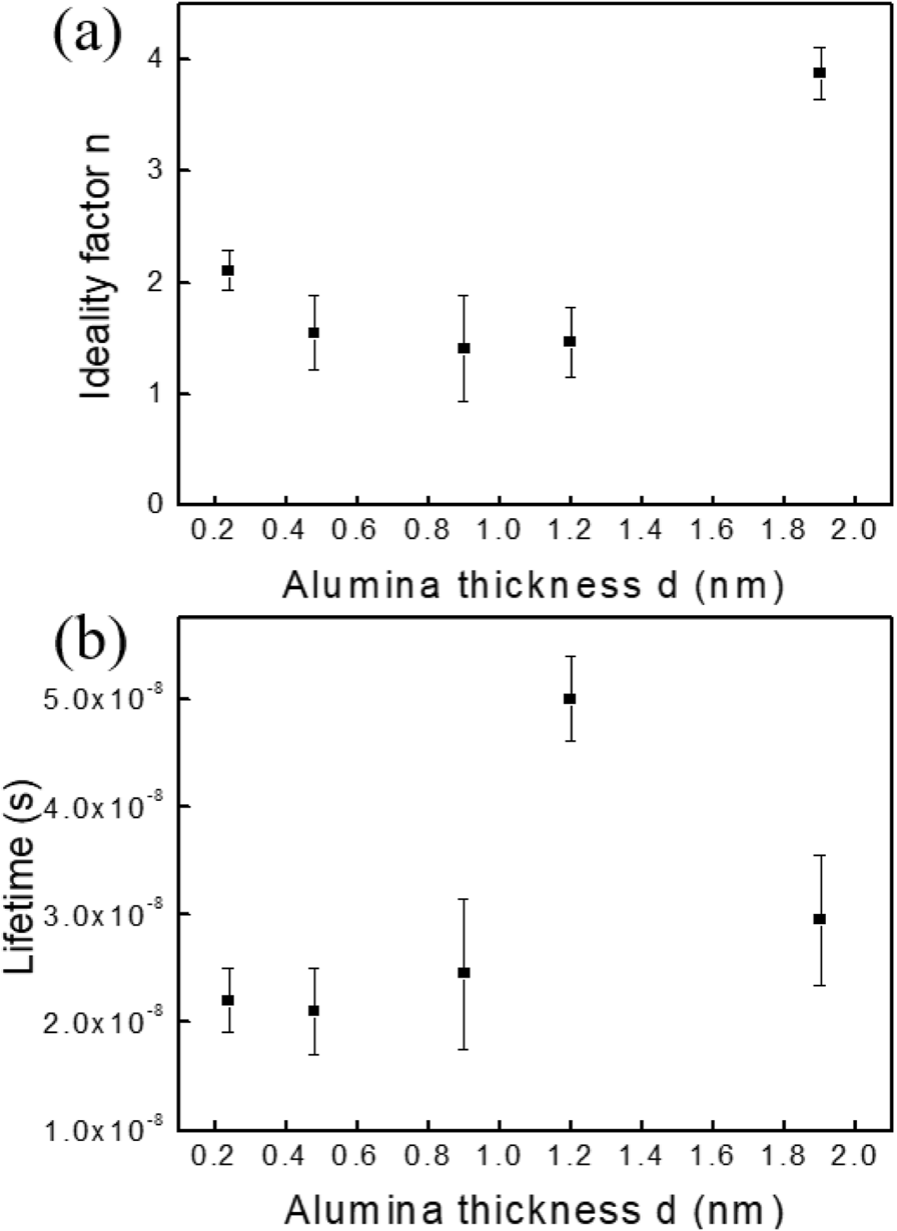
**a** Evolution of the ideality factor *n* versus the alumina thickness *d*. **b** Evolution of the lifetime *τ* versus the alumina thickness *d*

In the presence of a series resistance, the I-V curve of a solar cell is:

4 $$ I={I}_{\mathrm{L}}-{I}_0\ \exp \left(\frac{q\left(V+I\ {R}_{\mathrm{s}}\right)}{n\ k\ T}\right) $$

where *I* is the cell output current, *I*_L_ is the light generated current, *V* is the voltage across the cell, *T* is the temperature, *k* is the Boltzmann constant, *n* is the ideality factor, and *R*_S_ is the cell series resistance. For low injection level, with only band-to-band or Schottky Read-Hall recombinations, the ideality factor *n* is less than 2. It reaches the value of 1 when recombination is limited by minority carriers [31]. An increase of *n* indicates that an unusual recombination mechanism is taking place, involving both minority and majority carriers [32]. So, *n* is a signature of the recombination (or of the passivation) of the device. Moreover, *n* is also bound to *R*_s_ that increases the ideality factor [33]. The evolution of *n* versus the alumina thickness is shown in Fig. 3a.

For low thickness value (0.24 nm), *n* is greater than 2, which is the signature of unpassivated surface. As the alumina thickness increases, *n* decreases and stabilizes at about 1.5, evidencing an efficient passivation effect through the alumina. For an alumina thickness value of 1.9 nm, *n* increases abruptly to 4, accordingly to the abruptly increase of *R*_s_. So, both *R*_s_ and *n* show that the alumina layer well passivates the Si junction, associated with a low tunneling barrier. For *d* = 1.9 nm, the tunneling barrier increases, with a subsequent degradation of *R*_s_ and therefore of the ideality factor.

### Lifetime

Another key parameter which illustrates the passivation effect is the lifetime of the photocarriers, *τ*. Indeed, the lifetime is directly associated with the recombination rate of the carriers, bound to the concentration of surface defects (recombination centers). It was deduced from the measured open-circuit voltage, its time derivative, and the actual illumination level. Its evolution versus the alumina thickness is given in Fig. 3b. The evolution of the lifetime is in agreement with the previous results. The average value is low, mainly due to the unpassivated rear contact. However, it clearly exhibits an increase with the alumina thickness, accordingly to a better passivation of the front contact and with an optimum thickness value of 1.2 nm. For *d* = 1.9 nm, the lifetime decreases. It is possible that as the alumina thickness increases, less hydrogen diffuses from the SiN_x_ layer to the contact during the thermal annealing, and therefore, the chemical passivation effect decreases.

**Fig. 4:**
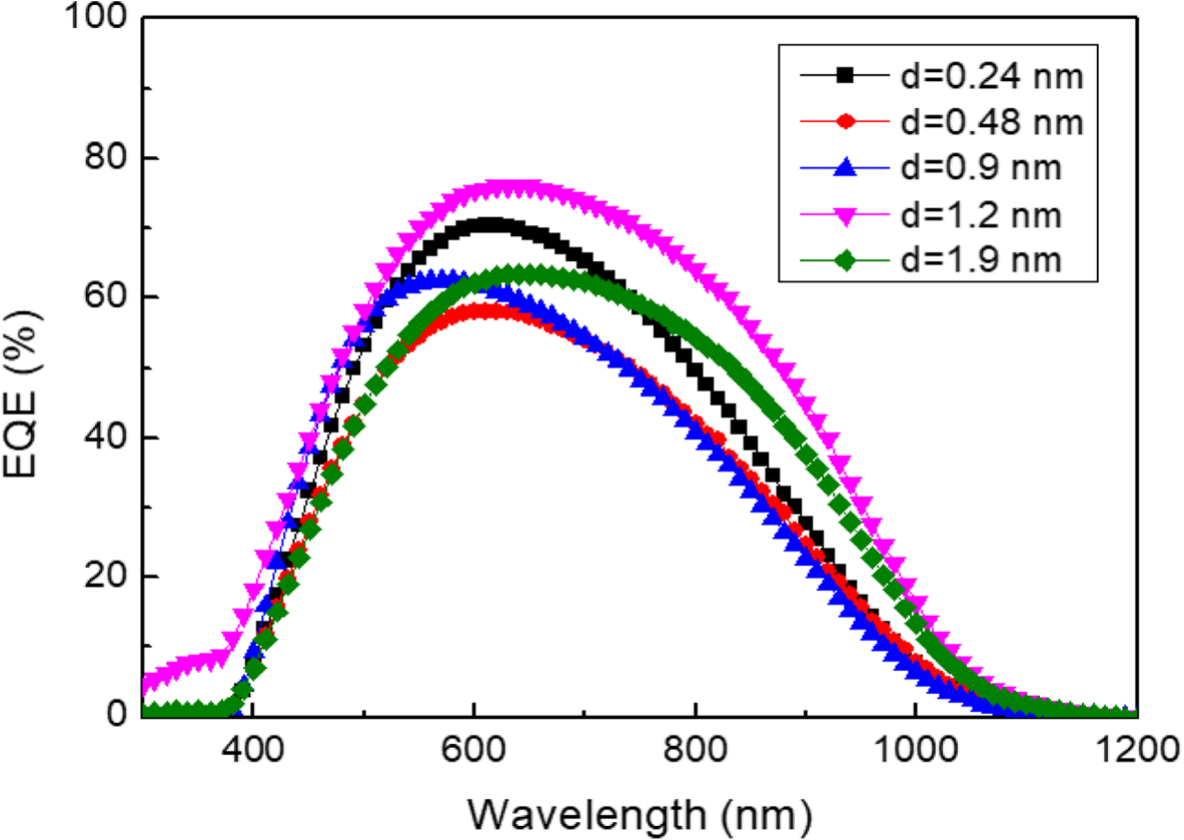
Measured EQE versus the wavelength for different alumina thicknesses

Figure 4 gives the measured EQE versus the alumina thickness. The best EQE is observed for *d* = 1.2 nm. The main improvement is observed for the wavelength varying from 600 to 900 nm. In all cases, the EQE is far from an ideal rectangular shape in the IR which is a signature of recombination at the unpassivated rear contact.

**Fig. 5:**
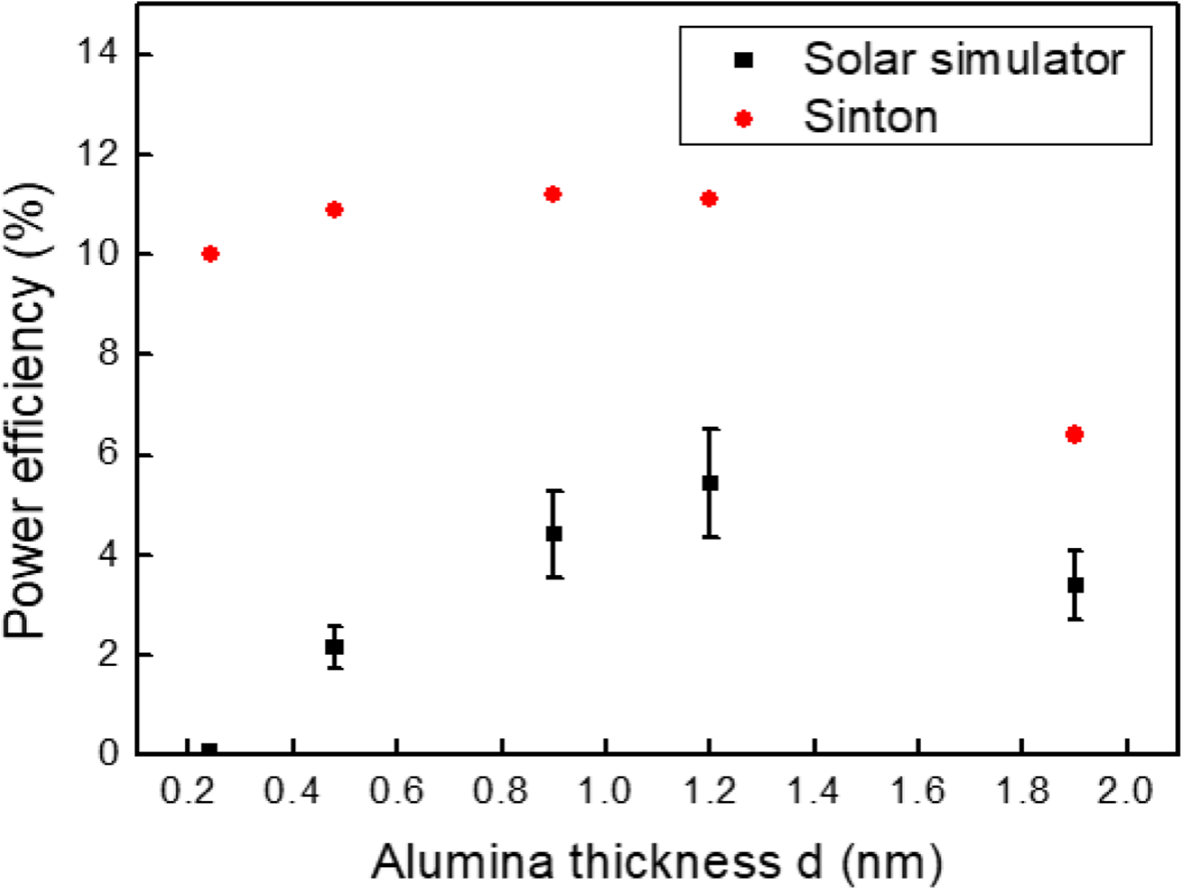
Measured and extrapolated power efficiency versus the alumina thickness *d*

In order to complete the analysis, we have studied the electrical parameters under illumination measured both under a solar simulator and using Sinton’s method. Figure 5 shows the power efficiency of the solar cells versus the alumina thickness: the measured one with the solar simulator and the optimized one without *R*_s_. As the alumina thickness increases, the measured PCE increases due to a better passivation effect reaching a maximum value of 5% for *d* = 1.2 nm, before decreasing for *d* = 1.9 nm. The corrected PCE for *R*_s_ = 0 has a quite constant value around 11%. This value is a reasonable one considering only the passivation of the rear contact. For *d* = 1.9 nm, the corrected yield decreases down to 6%, due to parasitic shunt resistance.

## Conclusions

Alumina deposited by ALD is an efficient method to passivate electrical contacts, a key parameter for silicon p-n junctions. In this work, tunneling atomic layer-deposited alumina layer with various thicknesses from 0.24 to 1.9 nm was used to passivate the implanted Si n^+^-p junctions. We have performed systematic HRTEM, STEM HAADF, and STEM EDX structural analyses correlated with a complete set of electrical measurements using both solar simulator and Sinton’s analyses. This original approach allows to claim that the optimum alumina thickness for achieving an efficient passivation effect is 1.2 nm. Although the device efficiency is not fully optimized in this work, the optimum alumina passivation could be beneficial for the development of the high-efficiency silicon-based solar cells.

## Abbreviations

ALD: Atomic layer deposition
EDX: Energy dispersive X-ray
EQE: External quantum efficiency
FIB: Focused ion beam
HAADF: High-angle annular dark field
HRTEM: High-resolution transmission electron microscope
PCE: Power conversion efficiency
PECVD: Plasma-enhanced chemical vapor deposition
RIE: Reactive ion etching
TEM: Transmission electron microscope
TMA: Trimethylaluminum
